# Morphological and physiological factors contributing to early vigor in the elite rice cultivar 9,311

**DOI:** 10.1038/s41598-020-71913-y

**Published:** 2020-09-09

**Authors:** Zai Shi, Tian-Gen Chang, Faming Chen, Honglong Zhao, Qingfeng Song, Mengyao Wang, Yanjie Wang, Zhiwei Zhou, Chongrong Wang, Shao-Chuan Zhou, Baoshan Wang, Genyun Chen, Xin-Guang Zhu

**Affiliations:** 1 National Key Laboratory for Plant Molecular Genetics, Chinese Academy of Science (CAS) Center for Excellence in Molecular Plant Sciences, Shanghai Institute of Plant Physiology and Ecology, CAS, Building No. 1, Room 402, Fenglin Road 300, Shanghai, 200032 China; 2 University of Chinese Academy of Sciences, Beijing, 100049 China; 3 CAS Key Laboratory of Computational Biology, Chinese Academy of Sciences-Max Plank Gesellschaft ()Partner Institute for Computational Biology, Shanghai Institute of Nutrition and Health, Shanghai Institutes for Biological Sciences, Chinese Academy of Sciences, Shanghai, 200031 China; 4 Huazhong Agricultural University, Wuhan, 430070 China; 5 Guangdong Provincial Key Laboratory of New Technology in Rice Breeding, Rice Research Institute, Guangdong Academy of Agricultural Sciences, Guangzhou, China; 6 Key Laboratory of Plant Stress Research, College of Life Sciences, Shandong Normal University, Ji’nan, 250014 Shandong China

**Keywords:** Photosynthesis, Plant breeding, Plant development, Plant physiology

## Abstract

Huanghuazhan (HHZ) and 9,311 are two elite rice cultivars in China. They have achieved high yield through quite different mechanisms. One of the major features that gives high yield capacity to 9,311 is its strong early vigor, i.e., faster establishment of its seedling as well as its better growth in its early stages. To understand the mechanistic basis of early vigor in 9,311, as compared to HHZ the cultivar, we have examined, under controlled environmental conditions, different morphological and physiological traits that may contribute to its early vigor. Our results show that the fresh weight of the seeds, at germination, not only determined the seedling biomass at 10 days after germination (DAG), but was also responsible for ~ 80% of variations in plant biomass between the two cultivars even up to 30 DAG. Furthermore, the 9,311 cultivar had a larger root system, which led to its higher nitrogen uptake capacity. Other noteworthy observations about 9,311 being a better cultivar than HHZ are: (i) Ten out of 15 genes involved in nitrogen metabolism were much more highly expressed in its roots; (ii) it had a higher water uptake rate, promoting better root-to-shoot nitrogen transfer; and (iii) consistent with the above, it had higher leaf photosynthetic rate and stomatal conductance. All of the above identified features explain, to a large extent, why the 9,311, as compared to HHZ, exhibits much more vigorous early growth.

## Introduction

Rapid population growth, decrease of arable land, and improved economic status globally, especially in developing countries, demand ~ 50% increase in crop productivity by 2030^1,2^. Rice is one of the major staple crops feeding about 3.5 billion people^3^; hence improvement of rice productivity is a major challenge for the solution of global food security^4^. With the rapid advances in functional genomics and in the genome editing technologies, molecular breeding or genome engineering for a particular feature is no longer a barrier facing us. Rather, the major challenge now is to define the optimal physiological and structural features for the desirable traits in our crops, including rice^5^. Once the superior features are identified, they can be incorporated through either traditional breeding, or marker assisted breeding or genome editing techniques^6–8^.

Early vigor, which refers to rapid emergence and early stage growth of plants, is a desirable trait for higher yields in crops, as it enables establishment of quick plant stand as well as its ground cover. An important indicator of early vigor is shoot biomass, or leaf area, since the latter is usually highly correlated with shoot biomass at the early growth stage^9–13^. Early vigor has several advantages. On the one hand, it makes plants much more competitive to other unwanted species, such as weeds^14–16^. On the other hand, early vigor enables faster establishment of the canopy, which helps shade the surface of the soil, increases the interception of solar energy and decreases soil water evaporation. These features, in turn, increase canopy photosynthesis and water use efficiency^17–19^. Due to these advantages provided to plants, early vigor has long been one of the selection criteria in various crop breeding programs, e.g. those in rice^20^, wheat^21^ and corn^22^.

Early vigor refers to plant growth of both the seedling as well as the post-seedling phase, with the former, being driven by seed reserves, while the latter mainly by photosynthesis and nitrogen uptake^23^. Before seed reserves are exhausted, factors controlling the quantity and remobilization efficiency of the reserve, as well as the growth efficiency, determine the speed of seedling development. As the endosperm provides most of the energy for seedling growth, the seed size is important; it has, indeed, been shown to be positively correlated with the rates of both seed germination as well as seedling growth in several species of plants^24^. Further, the total protein content in seeds has been correlated with seedling vigor in both wheat and maize^25^. We note that, in 241 F_10_ recombinant inbred lines of 5-day old rice seedlings^13^, total amylase and α-amylase activities, which control the rates of breakdown of starch in the endosperm, were positively correlated with shoot, root and total dry weight. Nevertheless, for different species and different varieties in the same species, the main factors controlling the seedling vigor are known to vary. For example, using natural variations of grain weight, measured on different panicles, and that too within the same rice cultivar, a positive correlation was observed between the seed weight and the seedling biomass after 14 days after germination (DAG)^26^. However, no correlation was found, in 27 different rice cultivars^27^, between the field vigor traits (e.g. percentage emergence, length and dry weight of shoots and roots) and the seed weight. Higher mitochondrial activity, indicating higher respiratory rate and greater amount of ATP generation, was identified as a major factor for seedling vigor in barley^28^, while in maize and soybean, no such difference was found between their vigorous and non-vigorous genotypes^29^. Thus, there is no consensus regarding what might be the key factor(s) controlling seedling vigor.

After the exhaustion of seed reserves, the photosynthetic capacity of the shoots becomes the major source for plant biomass accumulation, which, in turn, depends on the total light interception by leaves and leaf photosynthetic activity^18,30^. The photosynthetic capacity of the shoots is affected by many factors, including^18,31,32^: (i) the canopy architecture; (ii) the nitrogen content and its vertical distribution; (iii) the density and dynamics of stomata; and (iv) proteins involved in the “light reactions” and the carbon metabolism. Further, we note that the development of photosynthetically active leaves requires supply of nitrogen from the roots; in turn, the development of the shoots and the maintenance of their photosynthetic capacity affect the growth and function of roots^33–35^.

Although early vigor is a desirable trait for enhancing yield in direct-seeded rice^36^ and many quantitative trait loci (QTLs) for early vigor related traits in rice have been identified^13,37–41^, yet early vigor has not been effectively applied in the breeding programs. This is because, early vigor is a complex trait that is influenced by many different factors as mentioned above. Furthermore, dominant factors controlling early vigor are still poorly understood. Finally, early vigor may be closely linked with some undesirable traits, such as low grain quality, low harvest index or poor plant-type^13,37,42,43^. Thus, identification of key factors underlying early vigor in elite rice lines is expected to benefit future rice breeding programs.

An effective approach to identify key features controlling a desirable trait is through systematic comparative analysis^44^. Using this approach, we have identified key features that control canopy photosynthesis and thus biomass accumulation in two elite Chinese rice cultivars, Huanghuazhan (HHZ) and 9,311, during the entire growth season^45^. Since 2005, the rice cultivar HHZ has been one of the most popular rice in southern China since it has a high and stable yield, wide climate adaptation, and superior grain quality^46^. However, the 9,311 cultivar is one of the most important backbone parental lines for “super hybrid rice” in China^47^. At the tillering stage, the stronger early vigor of 9,311, over that of HHZ is a desirable trait, which gives these plants much higher canopy photosynthesis. Further, if this trait could be transferred into HHZ, its canopy photosynthesis, at the tillering stage, has been predicted to be doubled^45^. Therefore, the aim of this study is to determine the major features that contribute to strong early vigor in 9,311. Considering the difficulty of studying various root-related features, we have conducted early vigor trait measurements in a controlled hydroponic system, which were shown earlier to result in phenotypes correlated with that in the field and hence widely used in many studies^13,20,27,48–50^. We find that the strong early vigor of 9,311 can be largely attributed to a large seed weight, large root system and superior nitrogen and water uptake capacity of its root system.

## Results

### 9311 has stronger early vigor than HHZ under hydroponic condition

**Figure 1:**
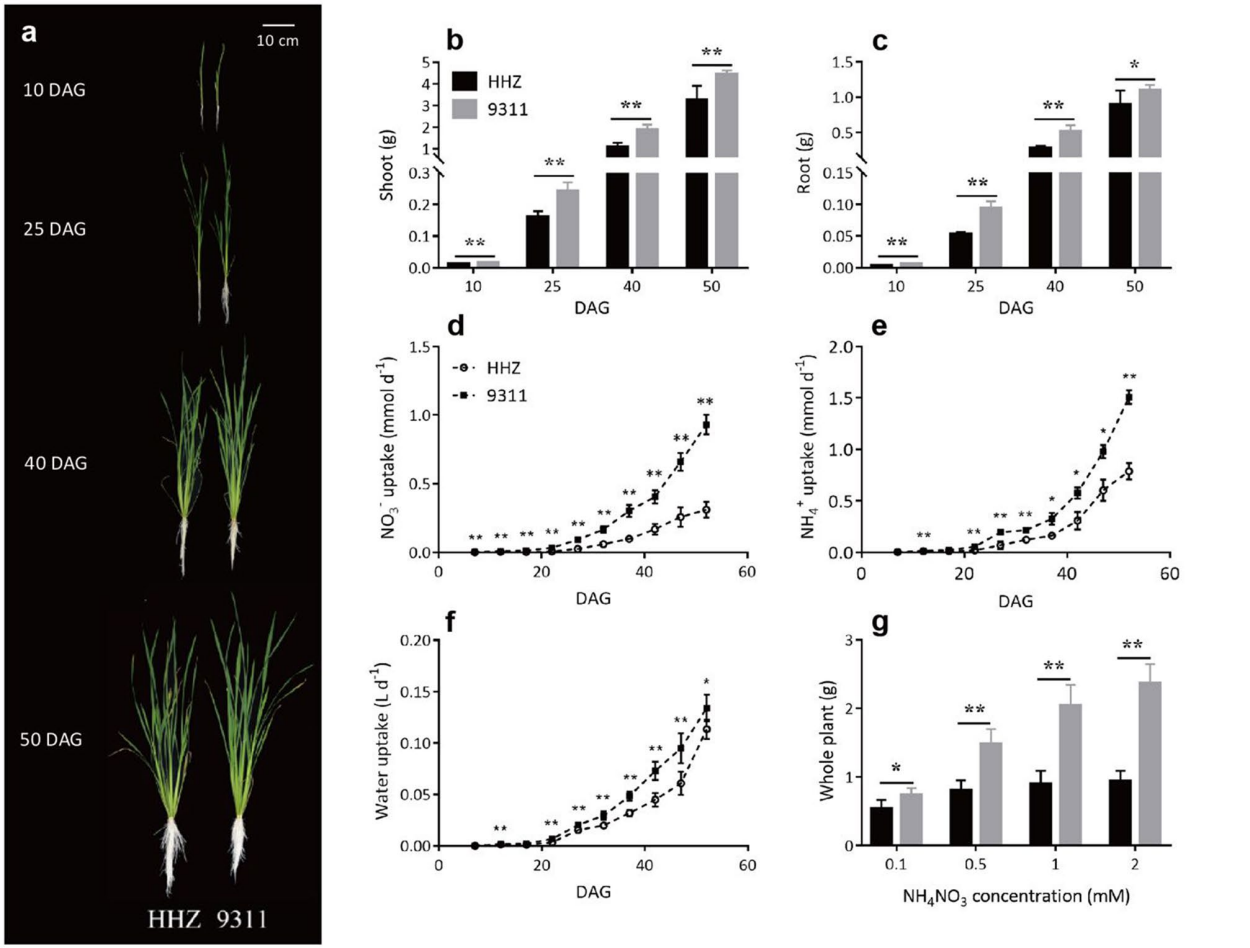
Comparison of morphological and physiological traits in HHZ and 9,311 during early growth. Plants (**a**), shoot dry weight (**b**) and root dry weight (**c**) at 10, 25, 40 and 50 DAG. Plant NO_3_^-^ uptake rate (**d**), NH_4_^+^ uptake rate (**e**) and water uptake rate (**f**) at 10, 25, 40 and 50 DAG. (**g**) plant biomass at 40 DAG under different solution NH_4_NO_3_ concentrations of 0.1, 0.5, 1 and 2 mM. Data presented are mean values with s.d. (n = 5 for panel **b**–**f**; n = 9 for panel **g**). *, **P < 0.05 and P < 0.01 (Student’s *t*-test), respectively.

Rice plants of 9,311 and HHZ were grown using a hydroponic system (see Supplementary Fig. S1 online) with 1 mM NH_4_NO_3_ from germination till 50 DAG (Fig. 1a). The accumulated biomass of 9,311 plants from seed to 50 DAG was 34% higher than that of HHZ. The shoot and the root dry weights of 9,311 were 44%, 49%, 79%, 37% and 67%, 78%, 84%, 22% higher than that of HHZ at 10, 25, 40 and 50 DAG, respectively (Fig. 1b,c). Further, the rate of NO_3_^-^ uptake by the 9,311 plants was always significantly higher than that of the HHZ plants from 10 to 50 DAG (Fig. 1d); and the rate of whole-plant NH_4_^+^ uptake of 9,311 became significantly higher than that of HHZ starting from 25 DAG (Fig. 1e). Specifically, the differences between the rates of nitrogen uptake of the two cultivars increased over time, which led to 199% and 92% higher NO_3_^-^ and NH_4_^+^ uptake rates at 50 DAG for 9,311, than that for HHZ (Fig. 1d,e). In addition, the 9,311 plants also had higher water uptake rate than the HHZ plants, especially from 25 DAG (Fig. 1f), which indicate higher bulk flow of water from the roots to the shoots. These results show that 9,311 has stronger early vigor over HHZ under hydroponic conditions; this includes increases in biomass accumulation, NO_3_^−^ uptake, NH_4_^+^ uptake, as well as water uptake.

Since nitrogen availability is an important factor influencing plant growth, the question arises as to whether the stronger early vigor of 9,311 over HHZ depends on environmental nitrogen availability. To answer this question, we cultured the two cultivars under four different NH_4_NO_3_ concentrations, i.e., 0.1, 0.5, 1 and 2 mM. The 9,311 plants had significantly higher biomass at 40 DAG under all nitrogen concentrations (Fig. 1g). However, the difference of biomass between 9,311 and HHZ at 40 DAG gradually decreased with the decrease of NH_4_NO_3_ concentration in the solution. Specifically, the biomass of 9,311 plants was 152%, 127%, 84% and 37% higher than that of HHZ under 2, 1, 0.5 and 0.1 mM NH_4_NO_3_, respectively (Fig. 1g). This indicates that nitrogen availability is an important factor contributing to the relative strength of early vigor in 9,311.

### Larger seed contributes to higher plant biomass of 9,311 plants

**Figure 2:**
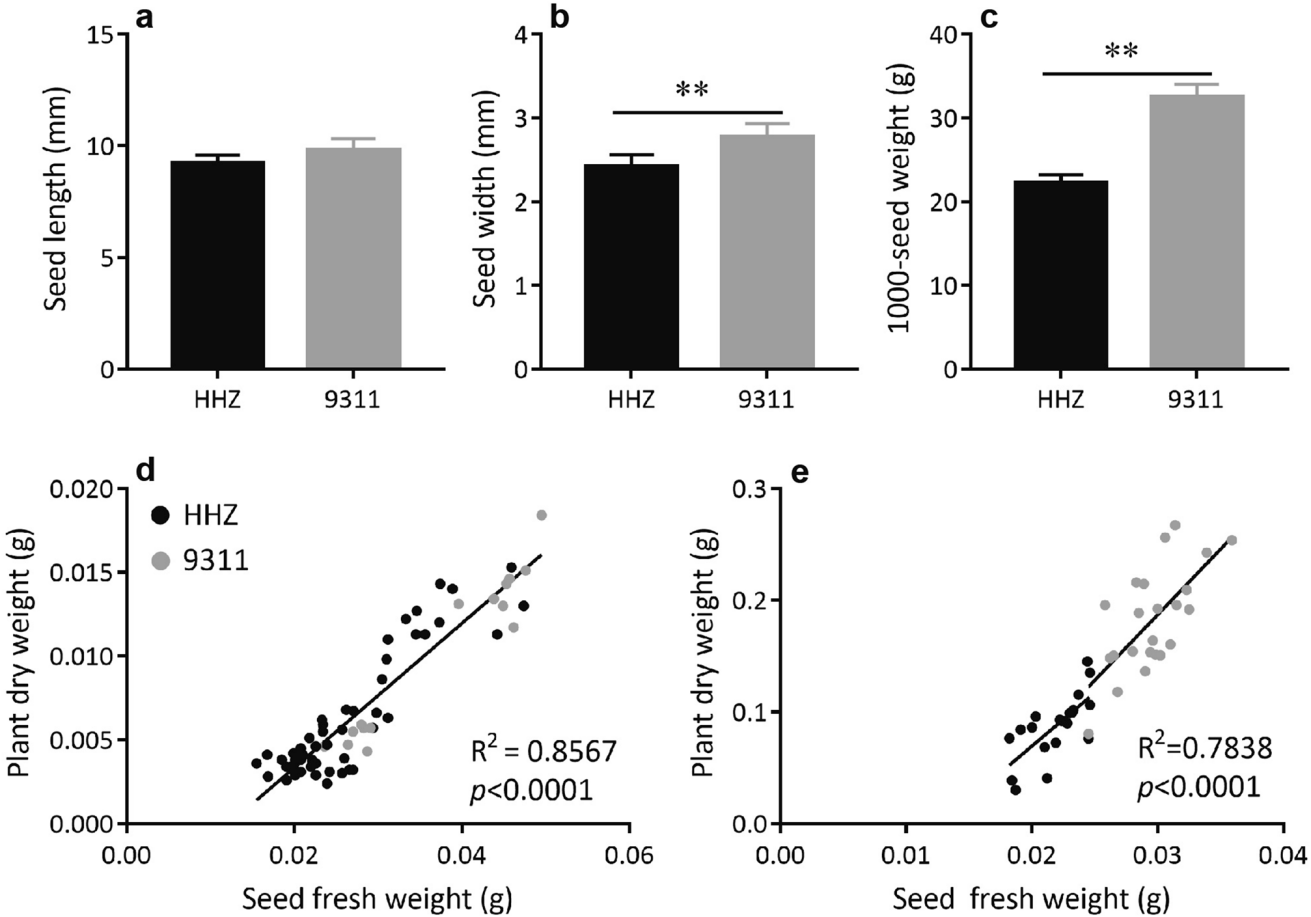
Seed size, seed weight and relationship between fresh weight of germinated seeds and plant biomass in HHZ and 9,311. Comparison of seed length (**a**), seed width (**b**) and 1,000-seed dry weight (**c**). Relationship between fresh weight of remaining part of cut germinated seeds and plant biomass at 10 DAG (**d**) or 30 DAG (**e**) in HHZ and 9,311. Data presented are mean values with s.d. (n = 5 for panel **a**–**c**; n = 68 for panel **d**; n = 42 for panel **e**). **P < 0.01 (Student’s *t*-test).

Although there were no significant differences in seed length between the 9,311 and HHZ plants, the former had significantly higher seed width and 1,000-grain weight than the latter (Fig. 2a–c). To test whether this trait is another contributing factor for stronger early vigor of 9,311 over HHZ, we removed some endosperm from the germinated seeds; this was done with a razor blade before the remaining seeds were weighed and put into the nutrient solution. We then measured the dry weight of shoots and roots that had developed from the remaining seeds at 10 DAG and 30 DAG, respectively. At 10 DAG, the seedling biomass was tightly correlated with the fresh weight of the seeds at germination (R^2^ = 0.86; Fig. 2d), indicating that the seedling biomass at 10 DAG was largely determined by the amount of the original seed reserves. Surprisingly, a high correlation can still be observed between the fresh weight of the remaining seeds and the biomass of the plants at 30 DAG. Specifically, an overall 78% variation in plant biomass at 30 DAG can be explained by the observed variations in the fresh weight of the germinated seed (Fig. 2e), regardless of the genetic difference between the two cultivars.

### The plants of 9,311 cultivar have higher leaf photosynthetic capacity

**Figure 3:**
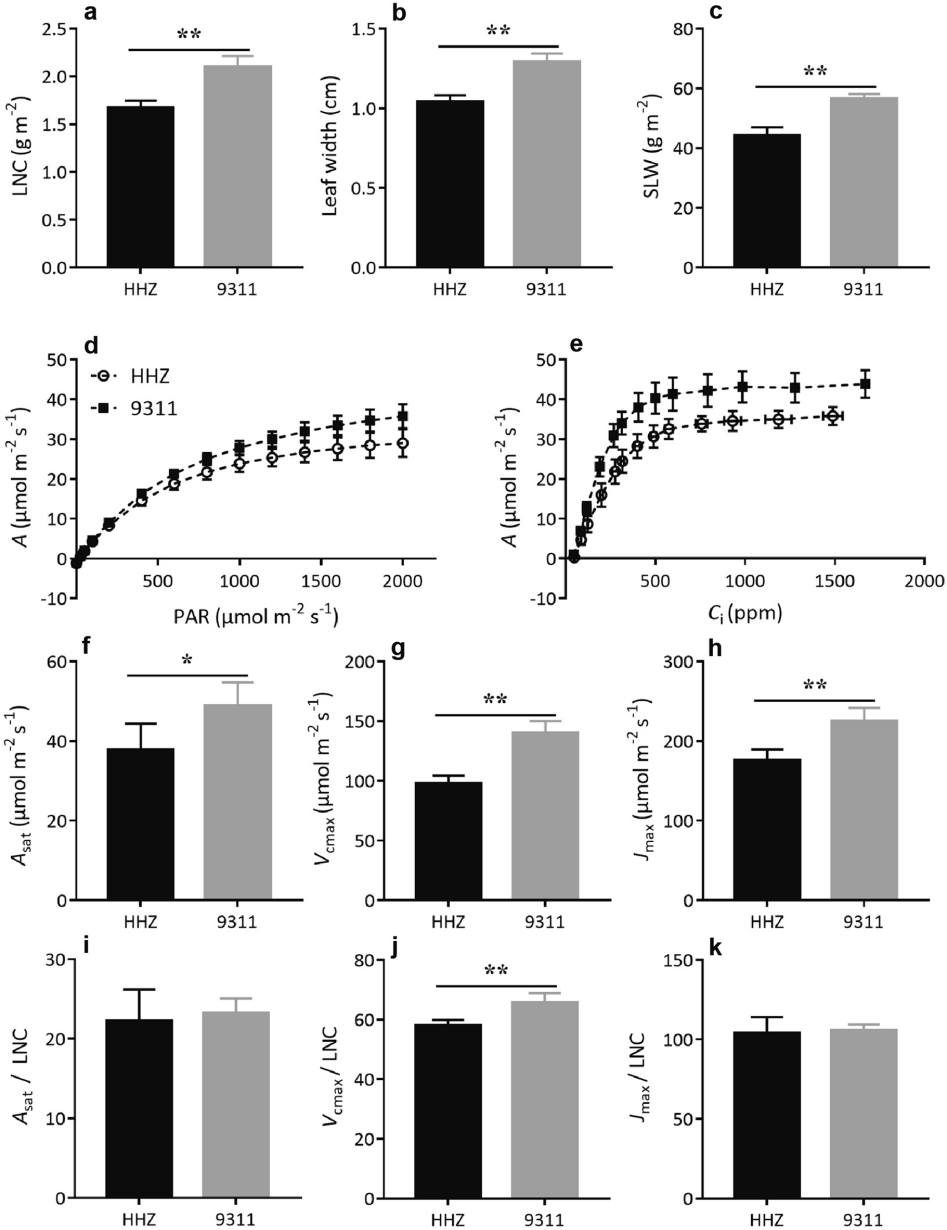
Comparison of leaf photosynthetic related parameters in 9,311 and HHZ at 50 DAG. The leaf nitrogen content (LNC; **a**), leaf width (**b**) and specific leaf weight (SLW; **c**) of the newest fully expanded leaves. The photosynthetic light response curves (**d**) and CO_2_ response curves (**e**). Light saturated photosynthetic rate extracted from the photosynthetic light intensity response curves (*A*_sat_; **f**), maximum rate of Rubisco carboxylation (*V*_cmax_; **g**) and maximum rate of electron transport for RuBP regeneration extracted from the photosynthetic CO_2_ concentration response curves (*J*_max_; **h**). *A*_sat_ normalized by LNC (**i**), *V*_cmax_ normalized by LNC (**j**) and *J*_max_ normalized by LNC (*J*_max_; **k**). Data presented are mean values with s.d. (n = 5). *, **P < 0.05 and P < 0.01 (Student’s *t*-test), respectively.

Since leaf photosynthesis is a major source of plant biomass accumulation during post-seedling growth, we measured leaf photosynthetic parameters of the two cultivars at 50 DAG. We note that 9,311 plants had 26% higher leaf nitrogen content (LNC) compared with HHZ (Fig. 3a). In addition,9,311 had 24% larger leaf width and 28% larger specific leaf weight (SLW, on an area basis), than that of HHZ (Fig. 3b,c). Moreover, comparisons of leaf photosynthetic light response curve and CO_2_ concentration response curve suggest a clear difference in leaf photosynthetic capacity between 9,311 and HHZ (Fig. 3d,e). Specifically, 9,311 had 30%, 53% and 34% higher rates of light saturated photosynthesis (*A*_sat_), Rubisco carboxylation (*V*_cmax_) and electron transport for RuBP regeneration (*J*_max_) than HHZ, respectively (Fig. 3f–h). However, there were no significant difference in *A*_sat_/LNC and *J*_max_/LNC between 9,311 and HHZ, whereas 9,311 had 13% higher *V*_cmax_/LNC than HHZ (Fig. 3i–k). In addition, the stomatal conductance of 9,311 was higher than that of HHZ under different light intensities, which resulted in a significantly higher intercellular CO_2_ concentration when light intensity exceeded 50 μmol photons m^−2^ s^−1^ (see Supplementary Fig. S2 online).

### The plants of 9,311 cultivar have a more vigorous root system

**Figure 4:**
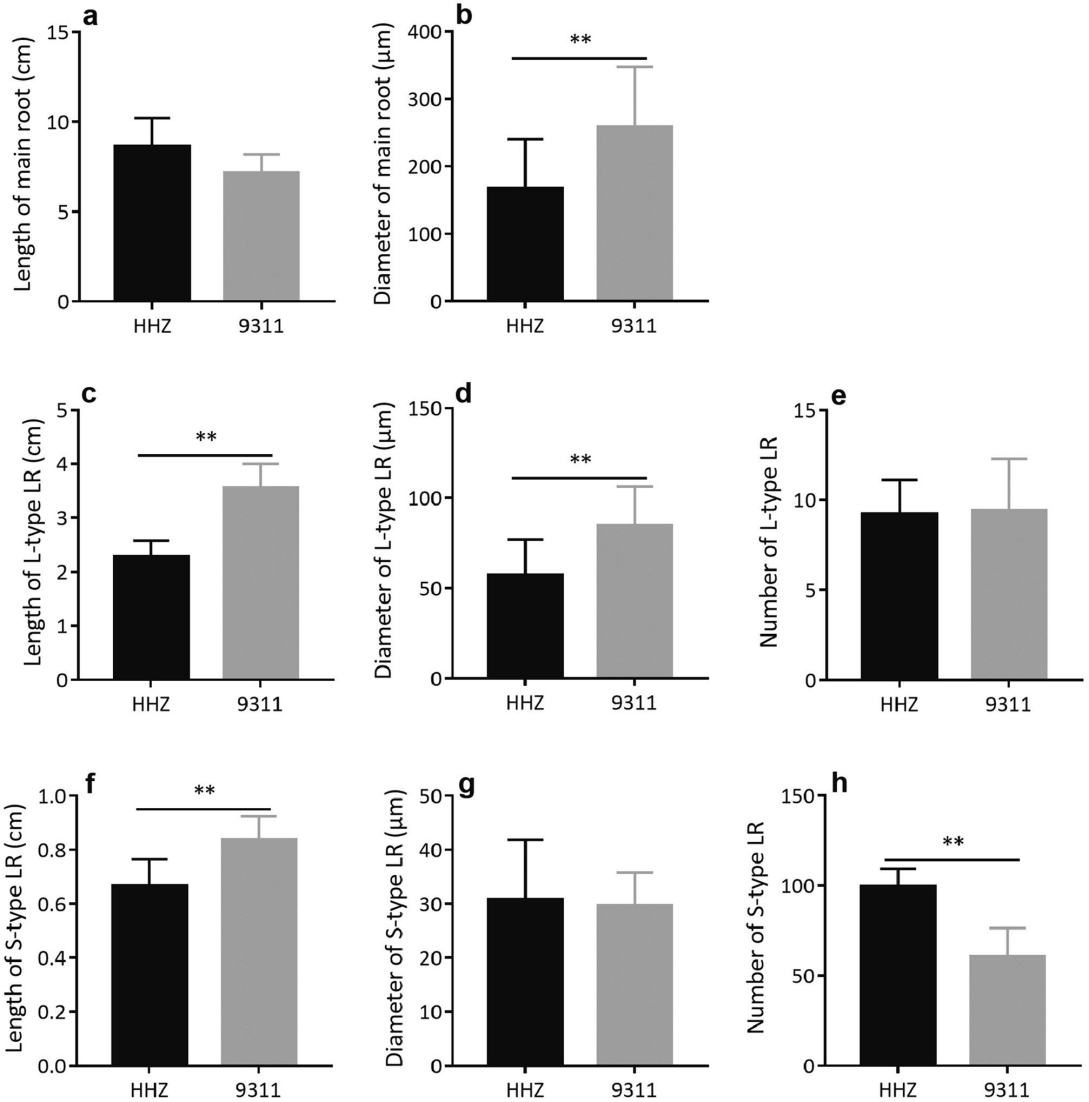
Comparison of morphological traits of the seminal root in HHZ and 9,311. The average length (**a**) and the diameter (**b**) of main roots at 10 DAG. The average length (**c**), the diameter (**d**) and average number (**e**) of L-type LRs at 10 DAG. The average length (**f**), the diameter (**g**) and average number (**h**) of S-type LRs at 10 DAG. Data presented are mean values with s.d. (n = 5). **P < 0.01 (Student’s *t*-test).

**Figure 5:**
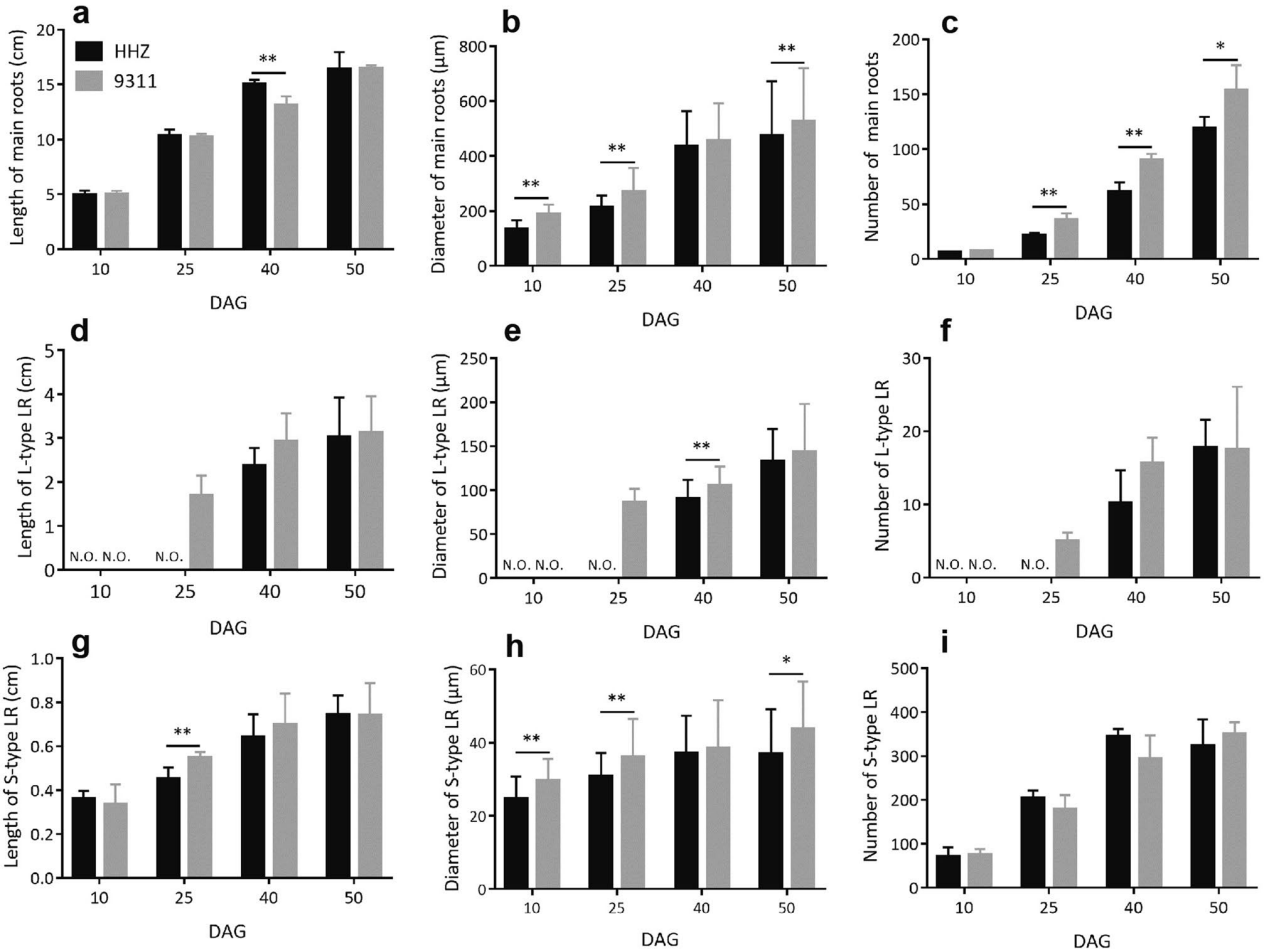
Comparison of morphological traits of crown roots in HHZ and 9,311. The average length (**a**), the diameter (**b**) and average number (**c**) of main roots at 10, 25, 40 and 50 DAG. The average length (**d**), the diameter (**e**) and average number (**f**) of L-type LRs at 10, 25, 40 and 50 DAG. The average length (**g**), the diameter (**h**) and average number (**i**) of S-type LRs at 10, 25, 40 and 50 DAG. Data presented are mean values with s.d. (n = 5). *, ** P < 0.05 and P < 0.01 (Student’s *t*-test), respectively. *NO* not observed.

Since the root is the main organ responsible for water and mineral nutrient uptake, we characterized the morphological characteristics of the roots in 9,311 and HHZ. The 9,311 plants not only had higher root dry weight throughout the 50 days early growth, as mentioned above, but also had a significantly higher root:shoot ratio than HHZ at 10 and 25 DAG (see Supplementary Fig. S3 online). At 40 and 50 DAG, there were no differences in root:shoot ratio between HHZ and 9,311 (see Supplementary Fig. S3 online). As the rice root system contains two types of main roots, i.e., the first emerging seminal root and the shoot-borne crown roots, and two types of lateral roots (LRs), i.e., branching L-type LRs which produce second-order LRs, and non-branching S-type LRs^51,52^, we made detailed measurements of length, diameter and number of these different types of roots. For seminal roots at 10 DAG, 9,311 had thicker main roots (Fig. 4b), longer S-type LRs (Fig. 4f), longer and thicker L-type LRs (Fig. 4c,d), whereas HHZ had a significantly larger number of S-type LRs (Fig. 4h). There were no significant differences in length of seminal roots (Fig. 4a), number of L-type LRs (Fig. 4e) and diameter of S-type LRs (Fig. 4g) between HHZ and 9,311 at 10 DAG. For crown roots, 9,311 developed a significantly larger number of roots from 25 DAG (Fig. 5c). Concurrently, 9,311 had thicker main roots and S-type LRs at most time points during early growth (Fig. 5b,h). Moreover, 9,311 developed L-type LRs earlier than HHZ, as 9,311 already had a number of L-type LRs at 25 DAG, a time when no L-type LR was observed in HHZ (Fig. 5d–f). The average length of crown roots (Fig. 5a), the number and average length of S-type LRs (Fig. 5g,i) were similar between HHZ and 9,311 at most time points during early growth.

**Figure 6:**
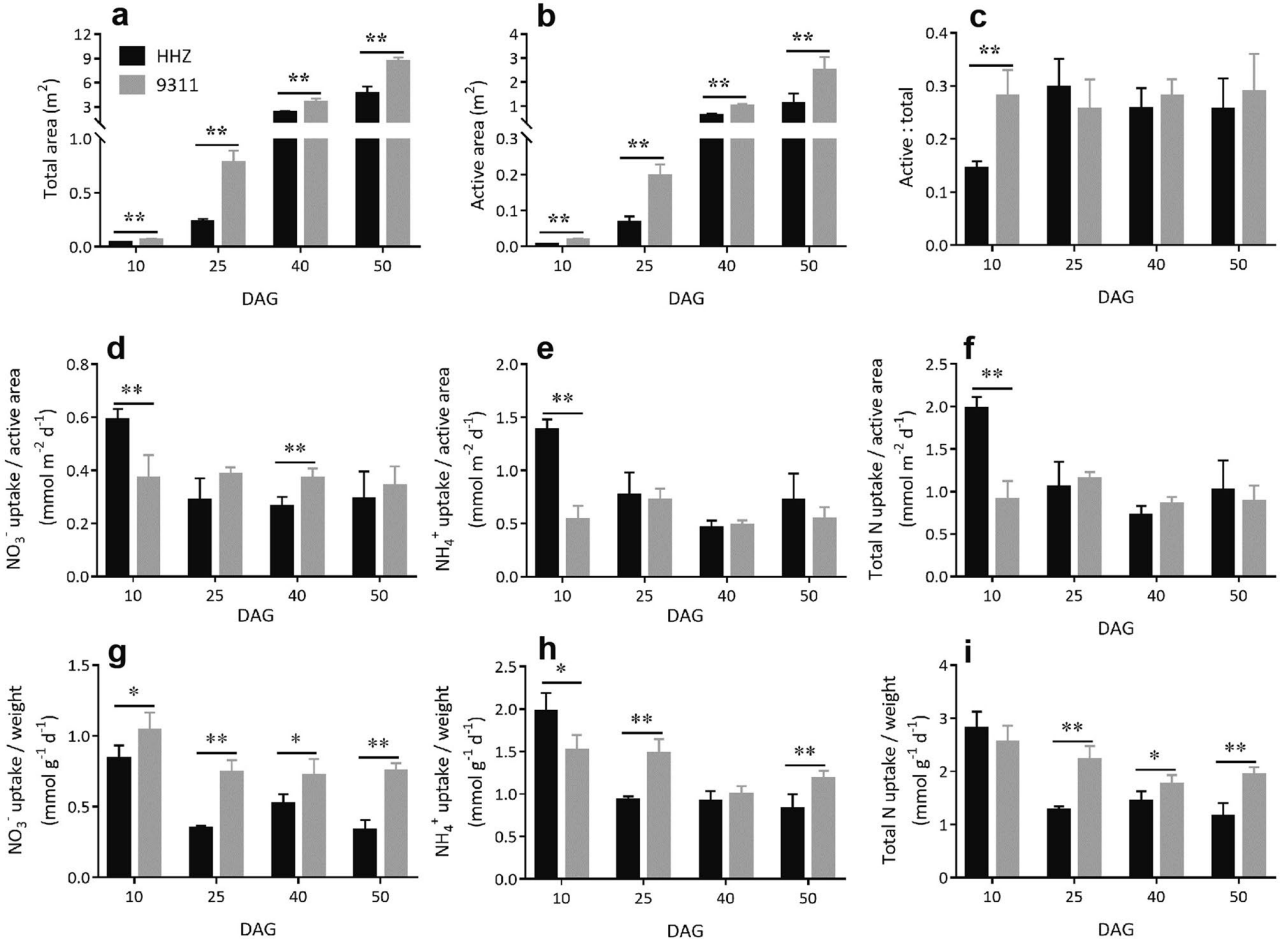
Comparison of root surface areas and normalized nitrogen uptake rate in HHZ and 9,311. Total root surface area (**a**), root active uptake surface area (**b**) and active-to-total root surface area ratio (**c**) at 10, 25, 40 and 50 DAG. Plant NO_3_^−^ uptake rate (**d**), NH_4_^+^ uptake rate (**e**) and total nitrogen uptake rate (**f**) on an active uptake area basis at 10, 25, 40 and 50 DAG. Plant NO_3_^−^ uptake rate (**g**), NH_4_^+^ uptake rate (**h**) and total nitrogen uptake rate (**i**) on a root dry weight basis at 10, 25, 40 and 50 DAG. Data presented are mean values with s.d. (n = 5). **P < 0.01 (Student’s *t*-test).

Together, seminal root, crown roots, L-type LRs, and S-type LRs accounted for larger total root surface area and active uptake area during early growth in 9,311 when compared to HHZ (Fig. 6a,b). Specifically, at 10 DAG, 9,311 had 77% and 244% larger total root surface area and active uptake area than HHZ, respectively. At 50 DAG, 9,311 had 87% and 126% larger total root surface area and active uptake area than HHZ, respectively (Fig. 6a,b). In addition to the total active uptake area of roots, nutrient uptake capacity on a unit area basis and on a unit biomass basis are two other indicators of root function. In this respect, both 9,311 and HHZ had a similar nitrogen uptake rate on an active surface area basis at all measurement time points except for 10 DAG (Fig. 6d–f), when the active-to-total root surface area ratio was much higher in 9,311 than that in HHZ (Fig. 6c). Whereas, on a root dry weight basis, 9,311 always had a much higher NO_3_^−^ uptake rate than HHZ (Fig. 6g); although 9,311 had lower NH_4_^+^ uptake rate at 10 DAG (Fig. 6h), both had similar total nitrogen uptake rate at 10 DAG, but after 10 DAG, 9,311, in comparison to HHZ, had significantly higher total nitrogen uptake rate (Fig. 6i). These results indicate that a larger root active uptake area must have contributed to the higher NO_3_^−^ and NH_4_^+^ uptake rate in 9,311, over than in HHZ (Fig. 1d,e).

### Differential gene expression of nitrogen metabolism in the roots of 9,311 and HHZ

**Figure 7:**
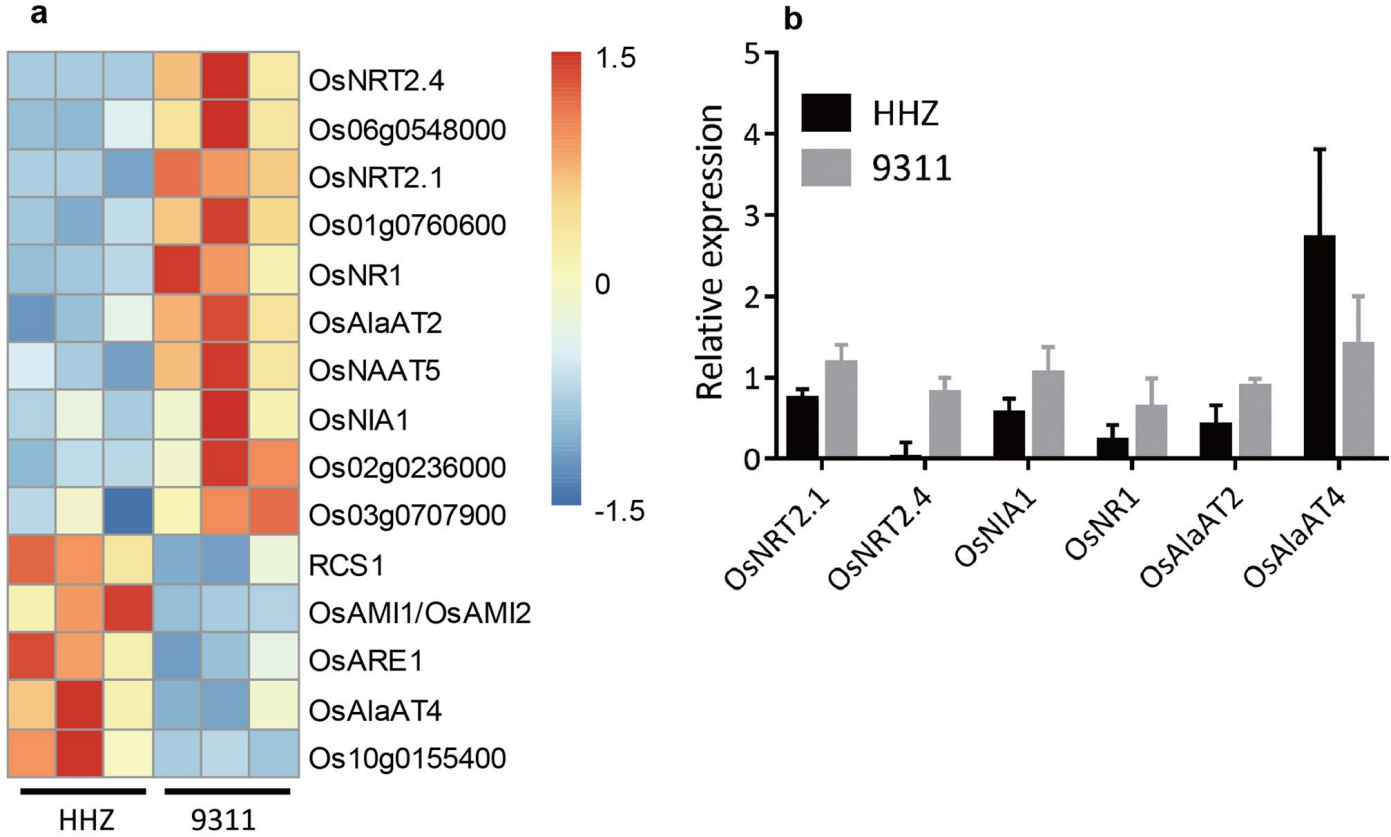
Differentially expressed genes related to nitrogen metabolism in the root of HHZ and 9,311 at 50 DAG. (**a**) differentially expressed genes related to nitrogen metabolism identified by RNA-seq and GO annotation of “nitrogen”, “nitrate” and “ammonia”. (**b**) Six differentially expressed genes validated by RT-qPCR. Data presented are mean values with s.d. (n = 3).

As root nitrogen metabolism also plays a crucial role in determining the overall root capacity, we compared the expression of genes related to nitrogen uptake and assimilation in the roots at 50 DAG between HHZ and 9,311. Nitrogen metabolism related genes were manually selected with the gene ontology (GO) term of “nitrogen” or “nitrate” or “ammonia” of significantly differentially expressed genes (DEGs) from the measured root transcriptome data (Fig. 7a). Of the 15 nitrogen metabolism related DEGs, 10 show significantly higher expression in the roots of 9,311, including two nitrate transporters (*OsNRT2.1* and *OsNRT2.4*), two nitrate reduction related genes (*OsNR1* and *OsNIA1*), and two amino-acid biosynthesis related genes (*OsAlaAT2* and *OsNAAT5*); on the other hand, five genes had significantly higher expression in the roots of HHZ, including two amino-acid biosynthesis related genes (*RCS1* and *OsAlaAT4*) and one potential negative regulator (*ARE1*) of nitrogen assimilation (Fig. 7a). Six of the differentially expressed genes were further confirmed using reverse transcription–quantitative PCR (RT-qPCR), they are *OsNRT2.1*, *OsNRT2.4*, *OsNIA1*, *OsNR1*, *OsAlaAT2* and *OsAlaAT4* (Fig. 7b).

## Discussion

Higher yield, higher resource use efficiency, higher stress tolerance, and better quality are major goals for future rice breeding^53–56^. However, the physiological and architectural features of rice plants that are crucial for realizing these goals are still far from being fully understood^5,57^. One approach to tackle this challenge is to systematically evaluate the available elite rice cultivars and identify their superior features, which can either be used as targets for mining molecular modules controlling those traits or be used directly as targets in current rice breeding programs. HHZ is one of the most popular inbred rice cultivars in southern China having stable grain yield and superior grain quality in the farmers’ field^46^. We have recently completed a systematic analysis of the different architectural and physiological properties of HHZ. Our results show that the better canopy architecture and higher leaf light-saturated photosynthetic rate between the panicle differentiation stage and the heading stage are main factors that contribute to its high radiation use efficiency^45^. However, compared to another elite cultivar, 9,311, HHZ had much lower early vigor, reflected by its lower leaf area index at the tillering stage^45^. Computational analysis suggests that if we could increase the early vigor in HHZ to the level of 9,311, namely, reaching the same leaf area index, LNC and canopy architecture as that of 9,311, a 135% increase in early-stage canopy photosynthesis could be achieved^45^. The 9,311 line is one of the elite parental lines for “super hybrid rice” in China^58–60^, Understanding what may contribute to early vigor of 9,311 is expected to help us in future rice functional genomics study and rice breeding, at least for the improvement of HHZ. Now, we discuss major features that might contribute to early vigor in 9,311. In the end, we will briefly comment on the potential factors to manipulate in HHZ for improvement of its early vigor.

We know that the endosperm is the major energy source for plant seedling growth^23^. Therefore, seed size is a dominant factor controlling the biomass of seedling, as already shown in several crop plants, e.g. rice, maize, and wheat^13,38,61,62^; whereas, its effect on post-seedling growth has hardly been studied. Here we show that the influence of seed weight on plant growth can last for a long time period, e.g. till 30 DAG (Fig. 2e). It is worth noting that by 30 DAG the plant biomass is already tenfold higher than the seed dry weight, majority of which is contributed by leaf photosynthesis and root nutrient uptake. These results indicate that seed size may not only affect seedling growth, but also have influence on post-seedling growth.

We know that the roots support plants’ nutrient and water uptake. Root mass, architecture and physiological properties are major factors controlling root’s ability to take up water and nutrients^63^. The 9,311 plants have significantly larger root mass and surface area after germination (Figs. 1c and 6a,b), which underlies their greater rate of NO_3_^−^ uptake (Fig. 1d), NH_4_^+^ uptake (Fig. 1e) and water uptake (Fig. 1f). The increased root mass and surface area in 9,311 are mainly attributed to a larger number of main roots, thicker main roots and LRs, longer L-type LRs on the seminal root, earlier development of L-type LRs on crown roots (Figs. 4 and 5). The root length density, which reflects root mass, was shown earlier to be positively correlated with the nitrate and water uptake capacity in maize, wheat and in several catch crop species^64–68^. Ni et al.^69^ have found that the rapid development of seedling root can explain competitive superiority of invasive plants. In addition, we know that the greater root length density is an important factor contributing to higher yield of the “super hybrid rice” cultivars compared to conventional elite varieties^70^. Although there are other studies showing that plants with a large root system may compromise shoot growth since these two compete for resources for growth^35,71–73^, here, we show that despite the higher root biomass and higher root:shoot ratio, 9,311 also had higher shoot production; further, no sign of growth penalty has been observed (Fig. 1b). In the future, more work is needed to identify the optimal assimilate partitioning pattern between the roots and the shoots for stronger early vigor. We emphasize that our current study was conducted with plants grown in a hydroponic system, which differs substantially from the field soil conditions. Therefore, the conclusions drawn here regarding root morphology between these two cultivars need to be further validated under the field conditions, using excavated roots from rice plants grown in the typical soil conditions used for growing rice.

In addition to the increased root mass, nutrient uptake and assimilation metabolism also plays a crucial role in determining the overall root capacity. Many genes related to nitrogen uptake and assimilation were differentially expressed in the roots of 9,311 and HHZ plants (Fig. 7). Specifically, *OsNRT2.1* and *OsNRT2.4*, which encode high-affinity and dual-affinity nitrate transporters, respectively, had significantly higher expression in the roots of 9,311 plants. Earlier, it was reported that knockout of *OsNRT2.4* decreased the number and length of LRs, and reduced total nitrogen uptake in rice^74^; while overexpression of *OsNRT2.1* increased rice nitrogen uptake and grain yield^75^. Concurrently, *OsNR1* and *OsNIA1*, two genes involved in catalyzing nitrate reduction^76,77^, which is a rate-limiting step in nitrate assimilation pathway, had significantly higher expression in roots of 9311plants. Interestingly, *OsARE1*, a gene recently identified to be a negative regulator of nitrogen use efficiency^78^, was higher expressed in the roots of HHZ plants. Whereas, in terms of *OsNRT1.1A* and *OsNRT1.1B* as two important genes controlling nitrogen use efficiency in rice^79,80^, *OsGRF4*^81^ as a major regulatory gene of nitrogen use efficiency, and *OsGA20ox1* as a candidate gene controlling seedling vigor in rice^82^, we, however, emphasize that none of these genes show differences in expression between HHZ and 9,311 cultivars (see Supplementary Fig. S4 online). All these results indicate that, in addition to its greater root mass and superior root architecture, 9,311 cultivar may also have higher metabolic activity of NO_3_^-^ uptake by its roots, as well as assimilation than the HHZ cultivars.

In addition to the known genes that may be manipulated, we also found a few genes whose function in nitrogen uptake and metabolism has not been fully studied. For example, we found that 9,311 had higher expression of *OsAlaAT2*, while HHZ had higher expression of *OsAlaAT4* (Fig. 7). Earlier, it was reported that *PnAlaAT3* might play an important role in nitrogen metabolism in poplar roots^83^. In rice, root specific expression of a barley AlaAT cDNA has been shown to increase nitrogen uptake, biomass and grain yield^84^. Detailed characterization of *OsAlaAT2* and *OsAlaAT4* are needed to clarify their roles in nitrogen uptake and assimilation. Further, we also found a number of other genes with GO annotation as “nitrogen” or “nitrate” or “ammonia” but without specific functions; these include Os06g0548000, Os01g0760600, Os02g0236000, Os03g0707900 and Os10g0155400 (Fig. 7). In addition, *AMI1*, *AMI2*, and *RCS1* are three other genes that show differential expressions, but their significance in nitrogen uptake and assimilation is less understood. Detailed functional studies on these genes may provide novel targets to manipulate root nitrogen uptake and assimilation capacity.

Finally, photosynthesis is the ultimate source of material and energy supporting growth and development of plants. In our research, presented here, we found that leaf photosynthetic capacity of 9,311 was higher than that of HHZ, as reflected by the higher *A*_sat_ (Fig. 3f), *V*_cmax_ (Fig. 3g) and *J*_max_ (Fig. 3h). We note that the LNC of 9,311 was about 26% higher than that of HHZ and the SLW was about 28% higher in 9,311 (Fig. 3a,c). As a result, the differences in LNC-normalized leaf photosynthetic parameters between 9,311 and HHZ are relatively small (Fig. 3i–k). The increased photosynthetic rate is mainly attributed, here, to increased LNC, which in turn might be related to higher SLW. This result is consistent with a previous outdoor pot experiment, where the increased leaf photosynthetic rate of rice cultivar Yuxiangyouzhan (25 days after sowing, compared to that of HHZ) was accompanied with higher leaf soluble protein level and higher SLW^85^, although there are also reports showing strong early vigor might result from a lower SLW, which means larger leaf area with the same leaf weight^30,71,86^.

In addition to the increased leaf photosynthetic capacity, 9,311 also had increased stomatal conductance (*g*_s_) as compared to HHZ (see Supplementary Fig. S2a online). This increased *g*_s_ can, indeed, increase intercellular CO_2_ concentration (see Supplementary Fig. S2b online) and hence photosynthetic CO_2_ uptake rate (Fig. 3d,f). Furthermore, higher *g*_s_ may also promote root-to-shoot nutrient transport, and stimulate root nutrient uptake^87–91^. Nonetheless, mechanisms underlying the increased *g*_s_ remain unknown. Higher water uptake capacity of 9,311 might have contributed to the increased *g*_s_, since increased water uptake capacity can help maintain higher turgor pressure and hence promote stomatal opening^92^.

**Figure 8:**
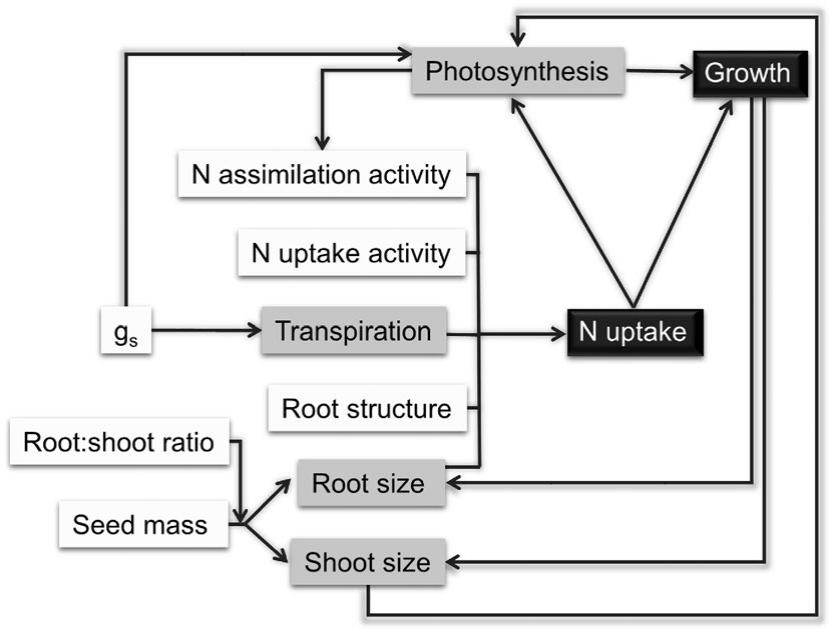
A brief diagram showing the major factors that contribute the difference in early vigor between the cultivar HHZ and the cultivar 9,311. The cultivar 9,311 has a larger seed mass, which helps establishment of a larger root mass and correspondingly a greater capacity of NO_3_^−^ uptake, NH_4_^+^ uptake, and water uptake. The increased nitrogen uptake and assimilation result in increased leaf photosynthetic capacity, which further increases the post-germination biomass accumulation.

In summary, in this study, we have examined different factors related to early vigor in two elite rice cultivars, 9,311 and HHZ (Fig. 8). Substantial differences in seed size/weight, root properties and leaf photosynthetic properties exist between these two cultivars. From systematic measurements and analysis, we conclude that the seedling vigor of 9,311 is mainly attributed to the large seed weight. After seedling establishment, greater root nitrogen uptake capacity of 9,311 increases the nitrogen content of leaves, leading to higher leaf photosynthetic capacity and further increase in post-seedling biomass. Therefore, potential options to improve early vigor of HHZ include increasing its seed weight, modifying the root architecture and engineering critical genes related to nitrogen uptake and assimilation. Although here we have identified several different factors affecting early vigor, their relative contribution or importance is still unknown and will be studied, in the future, by using transgenics or by physical manipulation, e.g. by removing parts of the lateral roots. Further studies on the function of the identified genes related to nitrogen uptake and assimilation, and on mechanisms underlying the increased stomatal conductance in the 9,311 cultivar may provide new targets to improve HHZ for increased early vigor as well.

## Materials and methods

### Plant material and hydroponic culture

Two elite rice cultivars HHZ and 9,311 were used for experiments. Dormancy of seeds was broken by incubation at 50 °C for 2 days. Germination was initiated in petri dishes with distilled water at 30 °C under dark for 4 days. The germinated seeds were transferred to mesh trays floating in culture solution in the growth chamber, with 96 plants per 1.4-l container (see Supplementary Fig. S1a online). Seedlings were then transferred to the greenhouse at 10 DAG, with 3 plants per 500-ml container. At 25 DAG, plants were transferred to 2-l containers, with 2 plants per container. Individual plants were arranged in 2-cm diameter holes drilled into container lids and fixed using sponge material wrapped around the stem (see Supplementary Fig. S1b online). Temperature in the greenhouse was set to 27 °C, and the relative humidity was 60–70%. Light was provided from 7 a.m. to 7 p.m., and the photon flux density (PFD) was about 400 μmol photons m^−2^ s^−1^. The culture solution was refreshed every 5 days. The composition of the culture solution was adapted from IRRI(International Rice Research Institute)’s protocol with 1 mmol L^−1^ NH_4_NO_3_, 0.6 mmol L^−1^ NaH_2_PO_4_·2H_2_O, 0.3 mmol L^−1^ K_2_SO_4_, 0.3 mmol L^−1^ CaCl_2_, 0.6 mmol L^−1^ MgCl_2_·6H_2_O, 50 μmol L^−1^ FeNaEDTA·3H_2_O, 50 μmol L^−1^ H_3_BO_3_, 9 μmol L^−1^ MnSO_4_·H_2_O, 0.3 μmol L^−1^ CuSO_4_·5H_2_O, 0.7 μmol L^−1^ ZnSO_4_·7H_2_O, 0.1 μmol L^−1^ Na_2_MoO_4_·2H_2_O, 7 μmol L^−1^ DCD-C_2_H_4_N_4_. The pH of the solution was 5.8–6.2.

For nitrogen gradient treatments, 4 different nitrogen concentrations were provided by changing the concentration of NH_4_NO_3_ in the culture solution to 0.1, 0.5, 1 and 2 mM, respectively. Plant shoots and roots were harvested at 40 DAG and dried separately for dry weight measurement.

### Endosperm removal procedure

For endosperm removal treatment, we first selected uniformly germinated seeds with 2–3 mm plumule, and then removed a random amount of endosperm from the germinated seeds with a razor blade. Then, the remaining seeds were put on an absorbent paper to remove excess water on the surface. After that, the remaining seeds were weighed and put into the nutrient solution cultured with the same protocol described above. Finally, about one half of the plants were randomly selected and harvested at 10 DAG, with the shoots and the roots dried separately for dry weight measurement; while the other half of the plants were harvested and measured at 30 DAG.

### Measurement of leaf gas exchange rate

Leaf photosynthetic light response curves (*A*-Q curves) and CO_2_ concentration response curves (*A*-*C*_i_ curves) were measured using the LI-6400 infrared analyzer (Li-Cor Inc, Lincoln, Nebraska, USA) at 50 DAG in the greenhouse. Specifically, for the measurement of *A*-Q curves, the reference CO_2_ concentration was set to 400 μmol mol^−1^, air flow rate was set to 500 μmol s^−1^, the block temperature was set to 26 °C and the initial light intensity was set to 1,800 μmol photons m^−2^ s^−1^. Leaves were first maintained under the condition, described above, for 20 min. Then we set an auto program to change the light intensity with the following sequence of PPFD: 2000, 1,800, 1,500, 1,200, 1,000, 800, 600, 400, 200, 100, 50, 25 and 0 μmol photons m^−2^ s^−1^. Leaves were maintained in the cuvettes for 2 min at each light intensity before photosynthetic rate was recorded. For the measurement of *A*-*C*_i_ curves, the light intensity was set to 1,800 μmol photons m^−2^ s^−1^, the air flow rate to 500 μmol s^−1^, the block temperature to 26 °C and the reference CO_2_ concentration to 400 μmol mol^−1^. The leaves were maintained under the above described condition for 20 min. Then, we set an auto program to change CO_2_ concentrations in the following sequence: 400, 350, 250, 150, 100 and 50 μmol mol^−1^. Leaves were maintained in the cuvette at each CO_2_ concentration for 2 min before photosynthetic rate was recorded. Next, the CO_2_ concentration was set back to 400 μmol m^−2^ s^−1^ and maintained at that concentration for 15 min. Finally, another loop was set in the auto program to change CO_2_ concentrations in the following sequence: 500, 600, 700, 900, 1,100, 1,400 and 1,800 μmol mol^−1^. The leaves were maintained in the cuvette at each CO_2_ concentration for 2 min before photosynthetic rate was recorded. The newest fully expanded leaves were used for measurements.

For parameter extraction from *A-C*_i_ curves, i.e., *V*_cmax_ and *J*_max_^93^, we followed the method used by Gu, et al.^94^. For parameter extraction from *A-*Q curves, i.e., *A*_sat_, we used a classical non-rectangular hyperbola model^95^.

### Measurement of root morphology

At 10, 25, 40 and 50 DAG, all roots for each plant were scanned by a scanner (V370 Photo, Epson). Then, the length of each main root, L-type LR, S-type LR and secondary LR were extracted manually using ImageJ (US National Institutes of Health, Bethesda, USA). The diameter of the main roots, L-type LRs, S-type LRs and secondary LRs were measured using a microscope equipped with a digital camera (SMART-biomicroscopy, Chongqing Optec Instrument Co., Ltd).

### Measurement of root volume and total and active uptake area

Root volume was measured by an Archimedes drainage method. Root total and active uptake area was measured with the methylene blue staining method^96^. In this method, it is assumed that the solute is attached onto the root surface first before it is absorbed by root. That is, when root is put in a methylene blue solution, at the beginning, a monolayer of methylene blue forms on the root surface uniformly; then, root surface with active uptake activity absorbs methylene blue, and restores the capacity to continue adsorbing more methylene blue molecules. Specifically, we first prepared three clean beakers and labeled them as #1, #2 and #3, and then added methylene blue solution to each of them. The volume of the methylene blue solution in each beaker was 10 times of the volume of the roots, and the concentration of the methylene blue solution was 0.064 g L^−1^. After the above procedure, roots were put on an absorbent paper to remove excess water, and were then put into the #1, #2 and #3 beaker successively for staining, with a staining time of 1.5 min in each beaker. Then, we measured the concentration of the methylene blue in each beaker with a spectrophotometer (Cary 50 Uv–visible Spectrophotometer, VARIAN, USA). Finally, the total and active uptake areas of roots were calculated as following:

1 $$ {\text{Total uptake area }}\left( {{\text{m}}^{{2}} } \right) \, = \, \left[ {\left( {{\text{C}}_{1}^{0} - {\text{C}}_{1}^{1} } \right)*{\text{V}}_{{1}} + \left( {{\text{ C}}_{2}^{0} - {\text{C}}_{2}^{1} } \right)*{\text{V}}_{{2}} } \right]*{1}.{1} $$

2 $$ {\text{Active uptake area }}\left( {{\text{m}}^{{2}} } \right) \, = \, \left( {{\text{C}}_{3}^{0} - {\text{C}}_{3}^{1} } \right)*{\text{V}}_{{3}} *{1}.{1} $$

in which C^0^_1_, C^0^_2_, and C^0^_3_ represent the original concentrations of the methylene blue in beaker #1, #2 and #3 before roots were put in, respectively; and C^1^_1_, C^1^_2_, and C^1^_3_ represent the concentrations of solution in beaker #1, #2 and #3 after root staining of the roots, respectively; V_1_, V_2_, and V_3_ represent the volume of the methylene blue solution in beaker #1, #2 and #3, respectively.

### Measurement of plant transpiration and root nitrogen uptake

At each time of culture replacement, the total volume of newly added solution in the pot was measured (V_1_), and 5 ml solution was sampled for the measurement of concentration of NH_4_^+^ (c_11_) and NO_3_^−^ (c_12_). At the same time on the next day, the total volume of solution in the pot was measured again (V_2_), and 5 ml solution was sampled for measurement of concentration of NH_4_^+^ (c_21_) and NO_3_^−^ (c_22_). To compensate the loss of NH_4_^+^ and NO_3_^−^ by other processes such as volatilization and denitrification, we further set a blank group of pots without plants. The total volume of solution (V_1_′; V_2_′) and concentration of NH_4_^+^ (c_11_′; c_21_′) and NO_3_^−^ (c_12_′; c_22_′) were measured on the day of culture replacement and the next day, respectively.

The concentration of NO_3_^−^ was measured by the High Performance Liquid Chromatography (HPLC, Agilent-1200, Whatman SAX—25 cm of HPLC column, mobile phase for 45 mM KH_2_PO_4_). The concentration of NH_4_^+^ was measured by ion chromatography (ICS-5000, THERMO). Finally, root NH_4_^+^ (v(NH_4_^+^)) and NO_3_^−^ (v(NO_3_^−^)) uptake rates were calculated, using the following equations:

3 $$ {\text{v}}\left( {{\text{NH}}_{{4}}^{ + } } \right) = \left[ {{\text{V}}_{{1}} *{\text{c}}_{{{11}}} - {\text{V}}_{{2}} *{\text{c}}_{{{21}}} - \left( {{\text{ V}}_{{1}}^{\prime } *{\text{c}}_{{{11}}}^{\prime } - {\text{V}}_{{2}}^{\prime } *{\text{c}}_{{{21}}}^{\prime } } \right)} \right]/{\text{N}}_{{\text{p}}} $$

4 $$ {\text{v}}\left( {{\text{NO}}_{{3}}^{ - } } \right) = \left[ {{\text{V}}_{{1}} *{\text{c}}_{{{12}}} - {\text{V}}_{{2}} *{\text{c}}_{{{22}}} - \left( {{\text{ V}}_{{1}}^{\prime } *{\text{c}}_{{{12}}}^{\prime } - {\text{V}}_{{2}}^{\prime } *{\text{c}}_{{{22}}}^{\prime } } \right)} \right]/{\text{N}}_{{\text{p}}} $$

where, N_p_ is the number of plants in the container. The transpiration rate (v(T)) was calculated as:

5 $$ {\text{v}}\left( {\text{T}} \right) = \left[ {{\text{V}}_{{1}} - {\text{V}}_{{2}} - \left( {{\text{V}}_{{1}}^{\prime } - {\text{V}}_{{2}}^{\prime } } \right)} \right]/{\text{N}}_{{\text{p}}} $$

### Measurement of sample dry weight and leaf nitrogen content

For sample drying, the newly harvested samples were put in an oven immediately. Temperature of the oven was first set to 110 °C for 1 h, and then switched to 70 °C for 3 days until the samples were completely dry. The dry weight of samples was measured with an electronic balance (JB / T, METTLER TOLEDO, USA). Ten centimeters of leaves that were used in photosynthetic rate measurements were harvested. For each 10 cm leaf segment, the width (*w*_L_, cm) was measured during photosynthetic rate measurement, the leaf dry weight (*m*_L_, g) was measured after the leaf was dried, and then leaf nitrogen concentration (*c*_N_, %) was measured with a vario ISOTOPE CUBE elemental analyzer (elementar, Germany). Finally, leaf nitrogen content (LNC; g m^−2^) was calculated as described below:

6 $$ {\text{LNC}} = c_{{\text{N}}} *m_{{\text{L}}} /\left( {{1}0*w_{{\text{L}}} } \right)*{1}0000 $$

### Analysis of expression abundance for genes related to nitrogen uptake and assimilation

At 50 DAG, plants were transferred to NH_4_NO_3_-free solution for 2 days. Then, plants were transferred back to normal culture solution (containing 1 mM NH_4_NO_3_) for 3 h. Then roots were sampled and frozen in liquid nitrogen (77 K) immediately. Three replications were used. We used the PureLink RNA Mini Kit (Life Technologies Corporation) for RNA extraction from roots. Firstly, we grinded the root sample with liquid nitrogen. Then, we followed the manufacture’s protocol for RNA extraction. The quality of purified RNA was assessed using an Agilent 2,100 Bioanalyzer (Agilent Technologies, Palo Alto, CA) and the RNA samples with RNA Integrity Number (RIN) higher than 6.8 were used for RNA library construction. RNA libraries were sequenced using the Ilumina X Ten platform in paired-end 150-bp mode, at the Novogene Company (China).

The adaptors of raw RNA-seq reads were trimmed using cutadapt software (https://cutadapt.readthedocs.io/en/stable/index.html) (Adaptor of read1: AGATCGGAAGAGCACACGTCTGAACTCCAGTCAC; Adaptor of read2: AGATCGGAAGAGCGTCGTGTAGGGAAAGAGTGT). The RNA-seq analysis was performed by the STAR software (version 2.6.1d)^97^ with the rice reference genome IRGSP-1.0 as well as GTF file (downloaded from Ensembl Plants https://plants.ensembl.org/). After generating genome index, the clean RNA-seq reads were aligned by STAR^97^ with “–quantMode GeneCounts” option to count number of reads per gene.

Differentially expressed genes were determined by the DESeq2 software^98^ with the read counts reported by STAR^97^. Only genes with the adjusted P-value below 0.05 were considered as differentially expressed genes.

The expressions of eight genes, i.e., *OsNRT2.1*, *OsNRT2.4*, *OsNIA1*, *OsNR1*, *OsAlaAT2* and *OsAlaAT4*, were further validated using reverse transcription-quantitative PCR (RT-qPCR). To do this, total RNA was first extracted from roots using the PureLink RNA Mini kit (Life Technologies Corporation). First-strand cDNA was synthesized by the TransScript One-step gDNA Removal and cDNA Synthesis SuperMix (TransGen Biotech Co.). Quantitative PCR was performed on the CFX connect Real-Time PCR Detection System (Bio-Rad) using the UNICONTM qPCR SYBR Green Master Mix (Yeasen Biotech Co.) following the manufacturer’s instructions. In each case, dissociation curves confirmed the purity of the amplified products. Finally, relative expression levels were calculated according to the 2^-△△CT^ method^99^, using OsActin1 as the internal control. The used primers for RT-qPCR are listed in Supplementary Table S1 online.

## Supplementary information

Supplementary file1

## Data Availability

The RNA-seq data discussed in this publication have been deposited in NCBI's Gene Expression Omnibus and are accessible through GEO Series accession number GSE148299 (https://www.ncbi.nlm.nih.gov/geo/query/acc.cgi?acc=GSE148299).
