# Supplementary material for: Morphological and physiological factors contributing to early vigor in the elite rice cultivar 9,311
Source: Sci Rep. 2020 Sep 9;10:14813. doi: 10.1038/s41598-020-71913-y (PMC7481250; doi:10.1038/s41598-020-71913-y)
Supplement: Supplementary file 1 — Supplementary file1 [file 41598_2020_71913_MOESM1_ESM.pdf]

Supplementary Information

Morphological and physiological factors contributing to early vigor in the elite rice cultivar 9311

Zai Shi[1#], Tian-Gen Chang[1#*], Faming Chen[2,3], Honglong Zhao[1,2], Qingfeng Song[1], Mengyao Wang[1,2], Yanjie Wang[1,2], Zhiwei Zhou[4], Chongrong Wang[5], Shao-Chuan Zhou[5], Baoshan Wang[6], Genyun Chen[1], Xin-Guang Zhu[1*]

Table S1. Primers of genes used for RT-qPCR in this study.

Gene	Gene ID	Primer	Sequence (5'-3')
OsNRT2.1	Os02g0112100	Forward	GAACAGGAGAAGAGCAAGGG
		Reverse	CAGAATTGTTTACGCCTTAGGC
OsNRT2.4	Os01g0547600	Forward	CAGCCTCCTCTGCACGCTCC
		Reverse	CATCATCGTCATCAACATCGC
OsNR1	Os08g0468100	Forward	ATGCACCACGGCTTCATCACCC
		Reverse	AAGCCGTTGACCAGCTCGTCCATG
OsNIA1	Os02g0770800	Forward	CTCAAGGTGTGGTACGTGG
		Reverse	GAGGTCATAGCCCATCTTCTC
OsAlaAT2	Os03g0183600	Forward	TCTTGATGAATCCTCAGGATGG
		Reverse	GTTGCTCATCAAGAATCTGTCC
OsAlaAT4	Os10g0390600	Forward	GATGTGTTCTATGCTCTCCGTCT
		Reverse	ATGCTTCATGGAACACATTAAAGCG

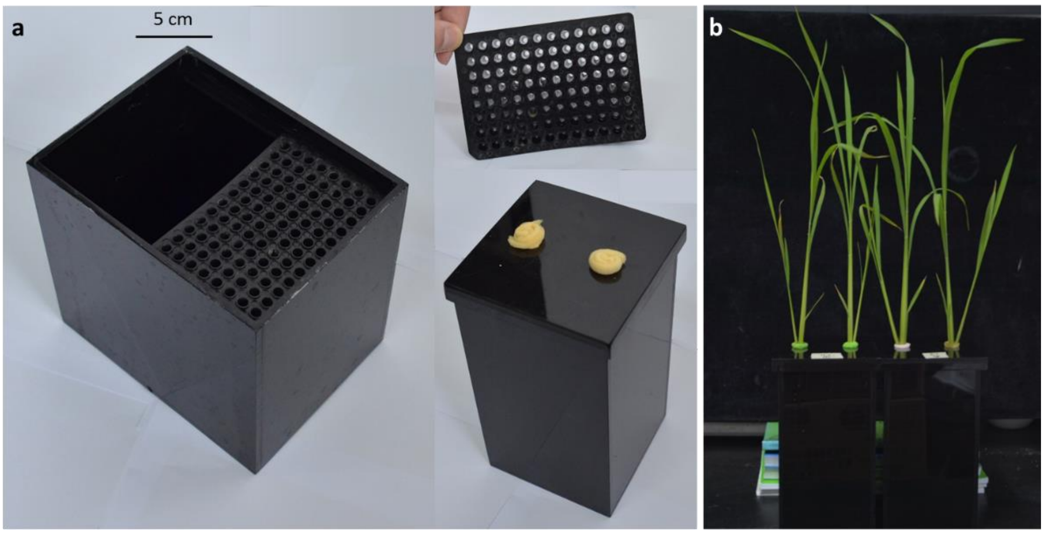

Figure S1. Photographs showing different views of a hydroponics system (**a**) and the working scenario when the hydroponic system is used to grow rice seedlings (**b**).

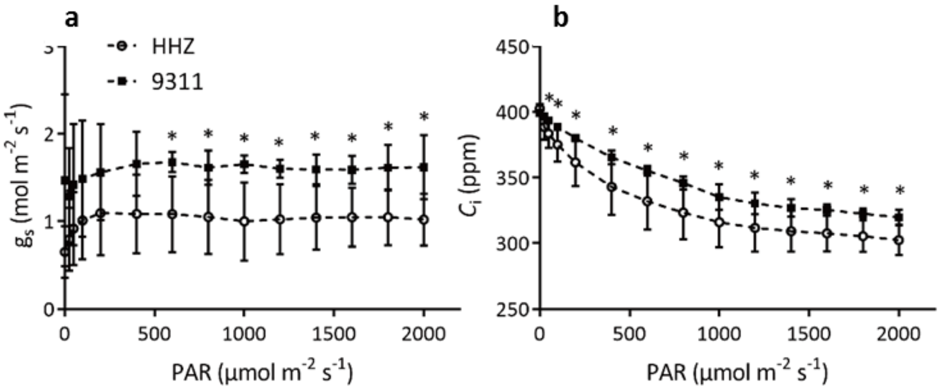

Figure S2. Stomatal conductance (g_s) and intercellular CO_2 concentration (C_i) of HHZ and 9311 in photosynthetic light intensity response curves. Data presented are mean values with s.d. (n = 5). * $P < 0.05$ (Student's *t*-test).

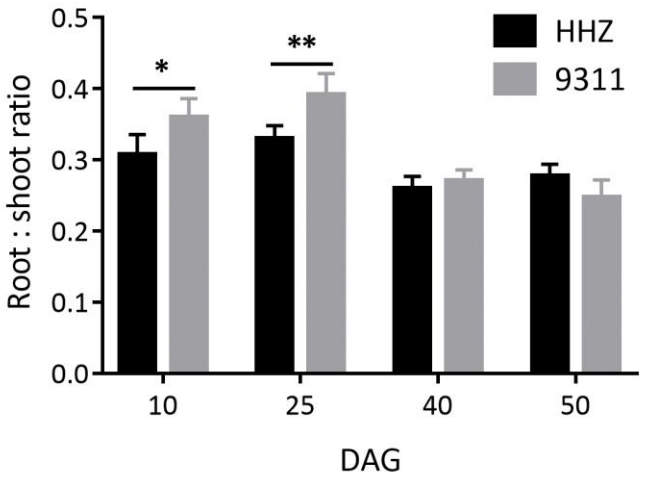

Figure S3. Root:shoot ratio of HHZ and 9311 at 10, 25, 40 and 50 DAG. Data presented are mean values with s.d. (n = 5). *, ** $P < 0.05$ and $P < 0.01$ (Student's *t*-test), respectively.

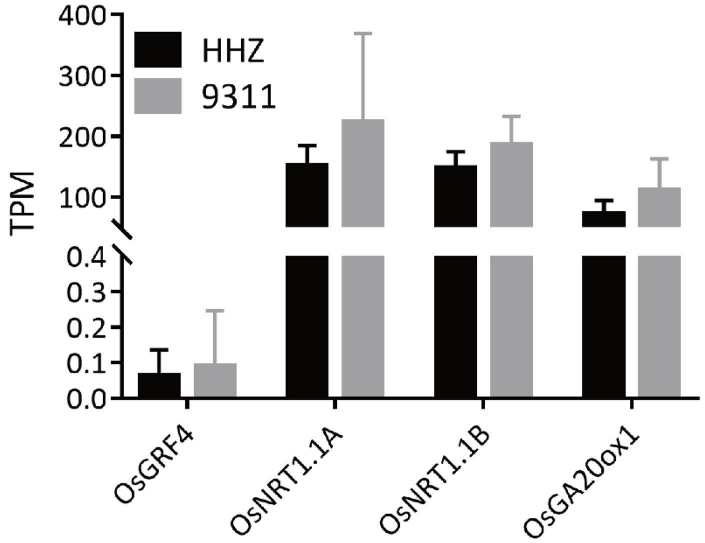

Figure S4. Relative expression of four important genes in nitrogen metabolism or early vigor in the root of 9311 and HHZ by RNA-Seq. Data presented are mean values with s.d. (n = 3).
